# A Comparison of Presolar Isotopic Signatures in Laboratory-Studied Primitive Solar System Materials and Comet 67P/Churyumov-Gerasimenko: New Insights from Light Elements, Halogens, and Noble Gases

**DOI:** 10.1007/s11214-023-00977-9

**Published:** 2023-05-24

**Authors:** Peter Hoppe, Martin Rubin, Kathrin Altwegg

**Affiliations:** 1 Max Planck Institute for Chemistry, Hahn-Meitner-Weg 1, 55128 Mainz, Germany; 2 Physikalisches Institut, University of Bern, Sidlerstrasse 5, 3012 Bern, Switzerland; 3 Center for Space and Habitability, University of Bern, Sidlerstrasse 5, 3012 Bern, Switzerland

**Keywords:** Comets, Meteorites, Isotopic compositions, Rosetta mission

## Abstract

Comets are considered the most primitive planetary bodies in our Solar System. ESA’s Rosetta mission to Jupiter family comet 67P/Churyumov-Gerasimenko (67P/CG) has provided a wealth of isotope data which expanded the existing data sets on isotopic compositions of comets considerably. In a previous paper (Hoppe et al. in Space Sci. Rev. 214:106, 2018) we reviewed the results for comet 67P/CG from the first four years of data reduction after arrival of Rosetta at the comet in August 2014 and discussed them in the context of respective meteorite data. Since then important new isotope data of several elements, among them the biogenic elements H, C, N, and O, for comet 67P/CG, the Tagish Lake meteorite, and C-type asteroid Ryugu became available which provide new insights into the formation conditions of small planetary bodies in the Solar System’s earliest history. To complement the picture on comet 67P/CG and its context to other primitive Solar System materials, especially meteorites, that emerged from our previous paper, we review here the isotopic compositions of H, C, and N in various volatile molecules, of O in water and a suite of other molecules, of the halogens Cl and Br, and of the noble gas Kr in comet 67P/CG. Furthermore, we also review the H isotope data obtained in the refractory organics of the dust grains collected in the coma of 67P/CG. These data are compared with the respective meteoritic and Ryugu data and spectroscopic observations of other comets and extra-solar environments; Cl, Br, and Kr data are also evaluated in the context of a potential late supernova contribution, as suggested by the Si- and S-isotopic data of 67P/CG.

## Introduction

Comets are considered the most primitive planetary bodies in our Solar System. They formed in the outer reaches of the protoplanetary disk at low temperatures and may thus have preserved significant amounts of the starting materials from which our Solar System formed some 4.57 Gyr ago, namely, interstellar dust, ices, and organics, provided post-formational thermal and aqueous alteration was kept minimal. This view is supported by recent models of planet formation by Neveu and Vernazza (2019). These models suggest that accretion of small bodies in the outer parts of the protoplanetary disk was completed only some 5-6 Myr after formation of calcium-aluminum-rich inclusions (CAIs), believed to be the first condensates in the Solar System. Consequently, heat production by decay of radioactive ^26^Al (half-life 716,000 yr), assumed to be the major heat source for melting and differentiation of planetesimals, would have been unimportant for comets.

Cometary matter has been studied in terrestrial laboratories for mineralogy and isotopic compositions. This includes matter from comet 81P/Wild 2, returned in 2006 by NASA’s Stardust mission (Brownlee et al. 2006), as well as anhydrous chondritic-porous interplanetary dust particles collected in the stratosphere (CP-IDPs; Ishii et al. 2008) and ultra-carbonaceous Antarctic micrometeorites (UCAMMs; Duprat et al. 2010) which are likely from comets. All these materials were shown to contain large amounts of presolar materials, among them so-called presolar grains (Zinner 2014) and organics with large isotopic anomalies in H and N (Busemann et al. 2006). Presolar grains are refractory, nanometer- to micrometer-sized dust grains which formed in the winds of evolved stars and in the ejecta of stellar explosions. Presolar grains carry large isotopic anomalies in their major, minor, and trace elements, the fingerprints of nucleosynthetic processes in their parent stars. Presolar (stardust) minerals include silicon carbide, graphite, silicon nitride, refractory oxides, and silicates. Most abundant among the presolar grains are the silicates which were found in matter from comet 81P/Wild 2 with concentrations of about 0.1%. Comparable abundances are observed in CP-IDPs (Floss and Haenecour 2016; Nguyen et al. 2022a). One CP-IDP, however, associated with comet 26P/Grigg-Skjellerup, was found to contain 1.5% presolar grains, the highest concentration of presolar grains found in extraterrestrial samples to date (Busemann et al. 2009). Isotopically anomalous stardust was estimated to have contributed a few percent to the interstellar dust in the molecular cloud from which our Solar System formed (Hoppe et al. 2017); the majority of interstellar dust, on the other hand, is assumed to have formed in the interstellar medium (ISM) (Zhukovska et al. 2008) and might have isotopic compositions similar to those of our Solar System which makes their identification difficult.

Presolar isotopic signatures are also present in certain types of meteorites. The most primitive meteorites are the carbonaceous chondrites. These meteorites contain presolar grains in concentrations of up to ∼0.02% (Floss and Haenecour 2016) and organics with large H- and N-isotopic anomalies (Busemann et al. 2006). Large enrichments in ^17^O and ^18^O in meteoritic cosmic simplectite (so-called COS phase) are interpreted to represent the O-isotopic signature of primordial water in the solar nebula (Sakamoto et al. 2007). The COS phase shows correlated mass-independent S and O isotopic signatures, which pinpoint the formation location of COS to the edge of the Solar System because the required UV irradiation was inferred to come from nearby massive stars (Vacher et al. 2021). Fujiya et al. (2019) have recently reported on relatively large ^13^C enrichments in carbonates in the Tagish Lake carbonaceous chondrite, probably the isotopic fingerprint of CO_2_ ice. This suggests that the Tagish Lake meteorite parent body, presumably a D-type asteroid, formed in the outer parts of the protoplanetary disk, beyond the orbits of Uranus and Neptune, or possibly even in the Kuiper belt. Similar conclusions were derived from studies of the carbonaceous chondrite Tarda which is related to the Tagish Lake meteorite (Marrocchi et al. 2021). All these observations clearly show that there is a link between matter from asteroids (meteorites) and comets.

In 2019 JAXA’s Hayabusa2 space mission returned samples from C-type asteroid Ryugu to Earth. Laboratory studies have shown that materials from Ryugu experienced extensive aqueous alteration and are mainly composed of materials similar to carbonaceous chondrites, particularly CI chondrites (Yokoyama et al. 2023). Like the Tagish Lake meteorite, carbonates in Ryugu show relatively large ^13^C enrichments (McCain et al. 2023). Despite the extensive aqueous alteration Ryugu contains presolar grains and organics with large H- and N-isotopic anomalies (Barosch et al. 2022; Nguyen et al. 2022b; Remusat et al. 2022), similar to observations for chondrites.

ESA’s recent Rosetta mission to Jupiter-family comet 67P/Churyumov-Gerasimenko (in the following 67P/CG) has expanded our knowledge about comets considerably. In an earlier paper (Hoppe et al. 2018) we reviewed the isotopic compositions of H, C, O, Si, S, Ar, and Xe obtained with the ROSINA (Rosetta Orbiter Spectrometer for Ion and Neutral Analysis) mass spectrometer (Balsiger et al. 2007) for the volatile component of 67P/CG, and of O and S in dust particles from the inner coma of 67P/CG measured with COSIMA (Cometary Secondary Ion Mass Analyzer) (Hilchenbach et al. 2016), and discussed these data in the context of respective meteorite data and other cometary data from spectroscopic observations. The D/H ratio of water in 67P/CG (Altwegg et al. 2015) falls at the upper end of what was observed in other comets and is distinctly higher than the D/H ratio of water in carbonaceous chondrites. This suggests that comet 67P/CG might be particularly primitive and might have preserved large amounts of presolar matter. This is also supported by hints for a possible late contribution from a nearby supernova (SN) to the formation site from Si- and S-isotopic compositions. This scenario, however, is not supported by the isotopic signatures of C and O of CO_2_, and of Xe.

Since our review paper was published in September 2018, in which we discussed the results for comet 67P/CG from the first four years of data reduction after arrival of Rosetta at the comet in the context of respective meteorite data, important new isotope data of several elements, among them the biogenic elements H, C, N, and O, for comet 67P/CG, the Tagish Lake meteorite, and C-type asteroid Ryugu became available, which provide new insights into the formation conditions of small planetary bodies in the Solar System’s earliest history. To complement the picture on comet 67P/CG and its context to primitive meteorites that emerged from the paper of Hoppe et al. (2018) we review here the isotopic compositions of H in the organic refractory dust from 67P/CG (Paquette et al. 2021), of H in methanol (Drozdovskaya et al. 2021), alkanes (Müller et al. 2022), and NH_3_ (Altwegg et al. 2019), of C, N, and O in various molecules (Schroeder et al. 2019; Biver et al. 2019; Altwegg et al. 2019, 2020; Müller et al. 2022), of the halogens Cl and Br (Dhooghe et al. 2017, 2021), and of the noble gas Kr (Rubin et al. 2018). These data are compared with the respective data of other primitive Solar System materials, in particular meteorites, and extra-solar environments; Cl, Br, and Kr data are also evaluated in the context of a potential late supernova contribution, as suggested by the Si- and S-isotopic data of 67P/CG. In addition, we will review the C-isotopic data of carbonates from the Tagish Lake meteorite (Fujiya et al. 2019) and Ryugu (McCain et al. 2023) and put them into context of C-isotopic data for 67P/CG.

The H, C, N, O, Cl, Br, and Kr isotope data for comet 67P/CG along with those of selected components in chondritic meteorites, IDPs, comet 81P/Wild 2, C-type asteroid Ryugu, the Sun, and extra-solar environments are listed in Table 1, reference values for isotopic compositions discussed here are given in Table 2.

**Table 1:** Hydrogen-, C-, N-, O-, Cl-, Br-, and Kr-isotopic compositions of distinct components in primitive meteorites, IDPs, comets 81P/Wild 2 and 67P/CG, the Sun, and extra-solar environments

Element	Component^1^	Isotopic Composition^2, 3, 4^		References
H		$\delta D_{\mathrm{VSMOW}}$ (‰)		
	Protosolar nebula	−870		[1]
	CCs/OCs bulk	−230 … +4400		[2-6]
	CCs/OCs organics	Up to +19000		[4, 7, 8]
	CCs/OCs H_2_O	−895 … +2800		[2, 4]
	IDPs/UCCAMs	−400 … +50000		[9, 10]
	81P/Wild 2	−240 … +2200		[11]
	Ryugu H_2_O^5^	59 ± 121		[55]
	PSC & PS CH_3_OH	Up to +640000		[12]
	**67P/CG H**_**2**_**O**	**+2220 ± 260**		**[13]**
	**67P/CG H**_**2**_**S**	**+2870 ± 970**		**[14]**
	**67P/CG CH**_**3**_**OH**	**+45000** **…** **+420000**		**[12]**
	**67P/CG alkanes**	**13590 ± 960**		**[13]**
	**67P/CG NH**_**3**_	**6060 ± 1280**		**[36]**
	**67P/CG refr. org.**	**8920 ± 3470**		**[15]**
C		$\delta ^{13} C_{\mathrm{PDP}}$ (‰)		
	Sun	−92		[17]
	CCs bulk	−30 … +25		[2, 3, 16]
	CCs/IDPs organics	−70 … 0		[7, 18]
	CCs carbonates	+20 … +90		[19, 20]
	81P/Wild 2 bulk	−18 ± 8		[21]
	Ryugu carbonates	+50 … +100		[56]
	Presolar SiC	−1000 … +70000		[22]
	ISM	−300 … +2600		[23, 24]
	Local ISM	+310 ± 290		
	**67P/CG CO**	**+34 ± 103**		**[25]**
	**67P/CG CO**_**2**_	**+59 ± 48**		**[26]**
	**67P/CG alkanes**	**−15 ± 57**		**[13]**
	**67P/CG CH**_**3**_**OH**	**−22 ± 107**		**[27]**
	**67P/CG H**_**2**_**CO**	**1225 ± 779**		**[27]**
N		$\delta ^{15}$N_Air_ (‰)		
	Sun	−383		[32]
	CCs bulk	−50 … +1100		[2, 3, 16, 28-31]
	CCs/IDPs/Ryugu org.	−380 … +4900		[7, 8, 34, 57]
	CAIs	−360		[33]
	81P/Wild 2	Up to +1100		[21]
	Presolar SiC	−1000 … +39000		[22]
	ISM	−500 … +2000		[35]
	**67P/CG NH**_**3**_	**1310 ± 490**		**[36]**
	**67P/CG NO**	**1170 ± 470**		**[36]**
	**67P/CG N**_**2**_ **+** **HCN**	**1090 ± 480**		**[36]**
O		$\delta ^{17}$O_VSMOW_ (‰)	$\delta ^{18}$O_VSMOW_ (‰)	
	Sun	−59	−59	[39]
	Bulk CCs	−6 … +9	−2 … +16	[37, 38]
	Bulk Ryugu	+10 ± 1	+19 ± 1	[58]
	CAIs, hibonites	−55 … 0	−55 … 0	[40-45]
	COS (H_2_O)	+180	+180	[46]
	81P/Wild 2 bulk	−11 ± 18	−11 ± 16	[21]
	Presolar Ox./Sil.	−900 … +120000	−1000 … 13000	[22]
	**67P/CG CO**_**2**_		**+10 ± 16**	**[26]**
	**67P/CG H**_**2**_**O**	**+121 ± 91**	**+122 ± 90**	**[13, 47]**
	**67P/CG O**_**2**_	**+710 ± 340**	**+450 ± 170**	**[27]**
	**67P/CG CH**_**3**_**OH**		**+7 ± 81**	**[27]**
	**67P/CG H**_**2**_**CO**		**+950 ± 760**	**[27]**
	**67P/CG SO**		**+1090 ± 450**	**[27]**
	**67P/CG SO**_**2**_		**+1010 ± 710**	**[27]**
	**67P/CG OCS**		**+800 ± 460**	**[27]**
	**67P/CG dust**		**−2 ± 60**	**[48]**
Cl		^37^Cl/^35^Cl		
	Bulk chondrites	0.3193 … 0.3197		[49]
	**67P/CG**	**0.336 ± 0.017**		**[54]**
Br		^81^Br/^79^Br		
	Bulk chondrites	0.9445 … 0.9826		[51]
	**67P/CG**	**0.95 ± 0.07**		**[50]**
Kr		Isotopic signature		
	Q	∼ normal (SW)		[52]
	P3 (diamond)	∼ normal (SW)		[52]
	HL (diamond)	enriched in ^86^Kr, depleted in ^80,82^Kr		[52]
	P6 (diamond)	∼ normal (SW)		[52]
	G (SiC)	enriched in ^82,86^Kr, depleted in ^80,83^Kr		[52]
	N (SiC)	∼ normal (SW)		[52]
	**67P/CG**	**depl. in** ^**83**^**Kr rel. to** ^**84**^**Kr**, $\boldsymbol{\delta^{83}\mathrm{Kr}_{\mathrm{SW}}= -81 \pm 34\permil}$		**[53]**

^1^CCs: Carbonaceous chondrites, org.: organics, COS: Cosmic symplectite, Ox/Sil: Oxides/Silicates. SW: Solar wind. PSC: Prestellar core. PS: Low-mass protostars. ^2^Errors are 1*σ*. ^3^$\delta ^{\mathrm{i}}\mathrm{X} = (({}^{\mathrm{i}}\mathrm{X}/{}^{\mathrm{ref}}\mathrm{X})_{\mathrm{sample}}/(({}^{\mathrm{i}}\mathrm{X}/{}^{\mathrm{ref}}\mathrm{X})_{\mathrm{standard}} - 1) \times 1000$; for standard ratios see Table 2. ^4^For presolar grains, the ISM, protostars, and 67P/CG $\delta D_{\mathrm{VSMOW}}$, $\delta ^{13} C_{\mathrm{PDP}}$, $\delta ^{15} N_{\mathrm{Air}}$, and $\delta ^{17,18}O_{\mathrm{VSMOW}}$ values were calculated from published D/H, ^12^C/^13^C, ^14^N/^15^N, and ^16^O/^17,18^O (or ^17,18^O/^16^O in case of presolar grains) ratios. ^5^Inferred from H in hydrous minerals.

[1] – Geiss and Gloeckler (2003); [2] – Alexander et al. (2012); [3] – Kerridge (1985); [4] – Alexander et al. (2010); [5] – Pearson et al. (2001); [6] – Yang and Epstein (1983); [7] – Alexander et al. (2007); [8] – Busemann et al. (2006); [9] – Messenger (2000); [10] – Duprat et al. (2010); [11] – McKeegan et al. (2006); [12] – Drozdovskaya et al. (2021); [13] – Müller et al. (2022); [14] – Altwegg et al. (2017); [15] – Paquette et al. (2021); [16] – Pearson et al. (2006); [17] – Hashizume et al. (2004); [18] – Floss et al. (2004); [19] – Fujiya et al. (2015); [20] – Fujiya et al. (2019); [21] – Stadermann et al. (2008); [22] – Zinner (2014); [23] – Wilson (1999); [24] – Milam et al. (2005); [25] – Rubin et al. (2017); [26] – Hässig et al. (2017); [27] – Altwegg et al. (2020); [28] – Franchi et al. (1986); [29] – Grady and Pillinger (1990); [30] – Ivanova et al. (2008) [31] – Sugiura et al. (2000); [32] – Marty et al. (2011); [33] – Meibom et al. (2007); [34] – Briani et al. (2009); [35] – Füri and Marty (2015); [36] – Altwegg et al. (2019); [37] – Clayton (2004); [38] – Lodders and Fegley (1998); [39] – McKeegan et al. (2011); [40] – Aléon et al. (2002); [41] – Fagan et al. (2002); [42] – Krot et al. (2002); [43] – Liu et al. (2009); [44] – Kööp et al. (2016a); [45] – Kööp et al. (2016b); [46] – Sakamoto et al. (2007); [47] – Schroeder et al. (2019); [48] – Paquette et al. (2018); [49] – Sharp and Draper (2013); [50] – Dhooghe et al. (2017); [51] – Wyttenbach et al. (1965); [52] – Ott (2014); [53] – Rubin et al. (2018); [54] – Dhooghe et al. (2021); [55] – Piani et al. (2023); [56] – McCain et al. (2023); [57] – Remusat et al. (2022); [58] – Yokoyama et al. (2023).

**Table 2:** Terrestrial or Solar System reference values for H-, C-, N-, O-, Cl-, Br-, and Kr-isotopic compositions

Isotope Ratio	Reference Material^1^	Reference Value
D/H	VSMOW	0.00015576
^13^C/^12^C	PDB	0.0112372
^15^N/^14^N	Air	0.0036765
^17^O/^16^O	VSMOW	0.0003799
^18^O/^16^O	VSMOW	0.0020052
^37^Cl/^35^Cl	SMOC	0.3196
^81^Br/^79^Br	NIST 977	0.9729
^80^Kr/^84^Kr	Solar wind	0.04120
^82^Kr/^84^Kr	Solar wind	0.20540
^83^Kr/^84^Kr	Solar wind	0.20340
^86^Kr/^84^Kr	Solar wind	0.30120

^1^Solar wind data from Ott (2014).

## Isotopic Compositions

### Hydrogen

**Fig. 1:**
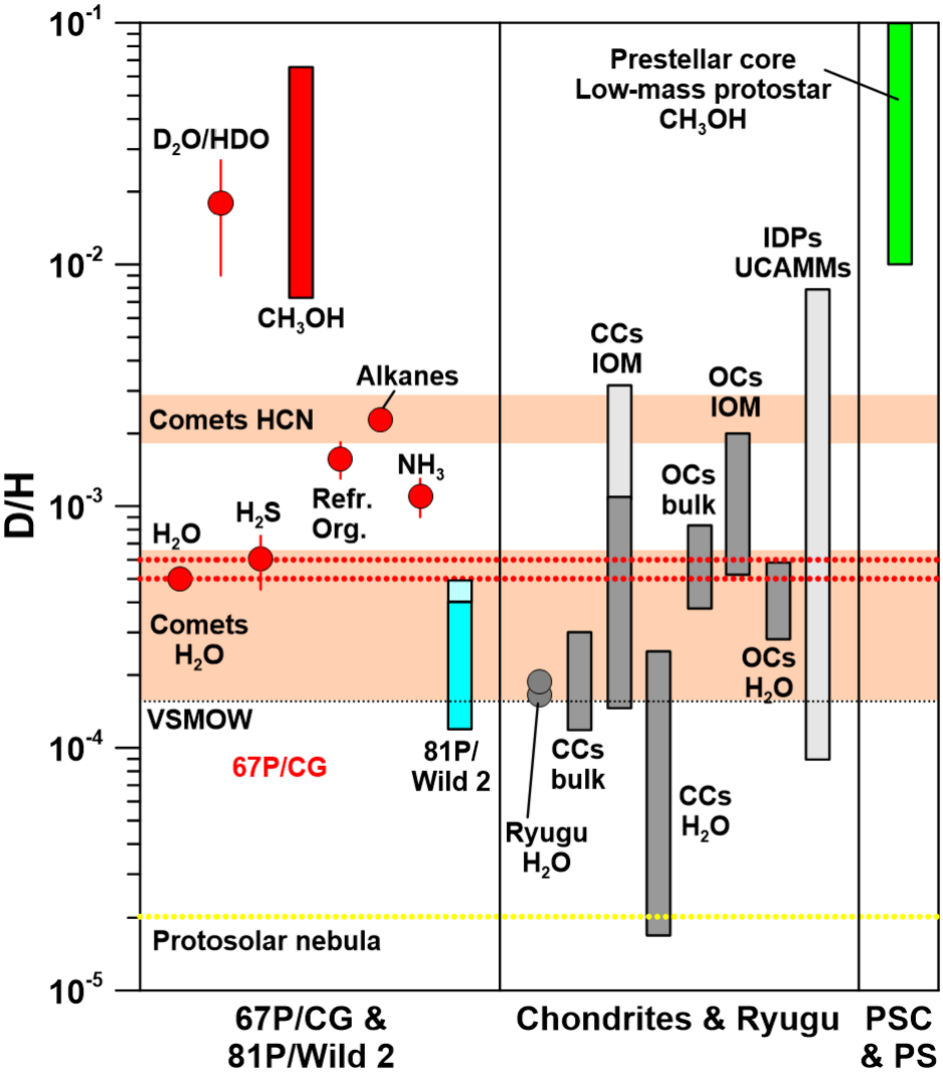
Hydrogen-isotopic compositions in various Solar System objects, prestellar cores (PSC), and low-mass protostars (PS). CCs: carbonaceous chondrites; OCs: ordinary chondrites; IOM: insoluble organic matter; VSMOW: Vienna standard mean ocean water. Data for 67P/CG are shown in red. The light-blue and light-grey shaded extensions in the bars for comet 81P/Wild 2, CCs IOM, and IDPs and UCAMMs indicate D/H ranges observed in micrometer-sized hotspots. The orange-shaded horizontal bars represent D/H ratios of water and HCN in 8 comets. Data sources: Protosolar nebula: Geiss and Gloeckler (2003); prestellar cores and low-mass protostars: See compilation of data in Drozdovskaya et al. (2021); 67P/CG: Altwegg et al. (2017, 2019), Drozdovskaya et al. (2021), Paquette et al. (2021), Müller et al. (2022); chondrites: Alexander et al. (2007, 2010, 2012), Busemann et al. (2006), Kerridge (1985), Pearson et al. (2001), Yang and Epstein (1983); IDPs and UCAMMs: Duprat et al. (2010), Messenger (2000); 81P/Wild 2: McKeegan et al. (2006); comets water and HCN: Biver et al. (2016), Bockelée-Morvan et al. (2015); C-type asteroid Ryugu (D/H in water inferred from D/H in hydrous minerals): Piani et al. (2023), Nittler et al. (2023). Errors are $1\sigma $. Figure adapted from Hoppe et al. (2018)

Hydrogen-isotopic compositions of various Solar System objects and of prestellar cores and low-mass protostars are displayed in Fig. 1. This is an updated version of the respective figure in Hoppe et al. (2018) in that new D/H data for various components in 67P/CG are added, namely, methanol (Drozdovskaya et al. 2021), alkines (Müller et al. 2022), and NH_3_ (Altwegg et al. 2019), measured with ROSINA, and refractory organics (Paquette et al. 2021), measured with COSIMA as well as D/H data for C-type asteroid Ryugu (Nittler et al. 2023; Piani et al. 2023).

As discussed in Hoppe et al. (2018), D/H ratios of H_2_O (Altwegg et al. 2015) and H_2_S (Altwegg et al. 2017) in 67P/CG, which show enrichments of D of about a factor 3.5 relative to terrestrial water, are higher than those of water in carbonaceous and, although less pronounced, ordinary chondrites (Alexander et al. 2010, 2012). An extended follow-up analysis of D/H data from 67P/CG by Müller et al. (2022) showed that this ratio is independent of cometary activity and heliocentric distance; the inferred D/H ratio from Müller et al. (2022) of $(5.01 \pm 0.40) \times 10^{-4}$ is slightly lower but compatible within uncertainties with the earlier ratio obtained by Altwegg et al. (2015). Piani et al. (2023) determined the D/H ratio of hydrous minerals in C-type asteroid Ryugu to $(1.65 \pm 0.10) \times 10^{-4}$, which can be considered a proxy of D/H in water at the time the hydrous minerals formed. It is a factor of 3 lower than the ratio for water in 67P/CG but fully compatible with the D/H ratio of hydrous minerals in CI chondrites (Piani et al. 2021, 2023). However, as pointed out by Nittler et al. (2023), inferred D/H ratios of hydrous minerals from many previous studies might be compromised by contamination from adjacent organics and terrestrial water. Nittler et al. (2023) inferred a D/H ratio of $(1.88 \pm 0.03) \times 10^{-4}$ for water in Ryugu, which is slightly higher than that of Piani et al. (2023) and that of CI chondrites. These authors used an experimental approach that minimizes the effect of H contamination from organics interspersed with silicates.

The D/H ranges observed for insoluble organic matter (IOM) in meteorites and of D-rich hotspots in IDPs and UCAMMs (Messenger 2000; Duprat et al. 2010) exceed the D/H ratio of H_2_O and H_2_S in 67P/CG (Fig. 1). The D/H ratio of methanol (CH_3_OH) in 67P/CG, a major volatile specie with an abundance of about 0.5% relative to water, was determined to lie between 0.0071 and 0.066 (Drozdovskaya et al. 2021), which is compatible with the D_2_O/HDO ratio (Altwegg et al. 2017) but more than an order of magnitude higher than the D/H ratios of H_2_O and H_2_S (Fig. 1). It is also higher than those of D-rich hotspots in IDPs and UCAMMs, i.e., the D/H signature of the methanol component in 67P/CG has not been observed yet in primitive planetary materials available for studies in terrestrial laboratories. Interestingly, the D/H ratio of methanol in 67P/CG is very close to those observed in cold (10-20 K) prestellar cores and low-mass protostars (Fig. 1; for references, see the compilation of data in Drozdovskaya et al. 2021). This suggests that cometary methanol stems from the prestellar core of the molecular cloud from which our Solar System formed. Strong D enrichments are also observed for the alkines methane (CH_4_), ethane (C_2_H_6_), propane (C_3_H_8_), and butane (C_4_H_10_) which, on average, have $\text{D/H} = (2.27 \pm 0.15) \times 10^{-3}$ which is about a factor of 4.5 higher than that of water in 67P/CG but comparable to D-rich hotspots in IDPs and UCAMMs (Fig. 1). This confirms observations from meteorites that organic molecules are more D-enriched than water (Fig. 1).

Refractory organics in 67P/CG have a mean D/H ratio of $(1.57 \pm 0.27) \times 10^{-3}$, as inferred from measurements of 25 dust particles (Paquette et al. 2021). This is about a factor of 3 higher than in water in 67P/CG (Fig. 1). As pointed out by Paquette et al. (2021) the relatively high D/H ratio of refractory organics in 67P/CG suggests that refractory carbonaceous matter in comet 67P/CG is less processed than the most primitive IOM in meteorites, which, without consideration of hotspots, has a D/H ratio in the range of about $10^{-4}$ to $2 \times 10^{-3}$ (Fig. 1).

### Carbon

**Fig. 2:**
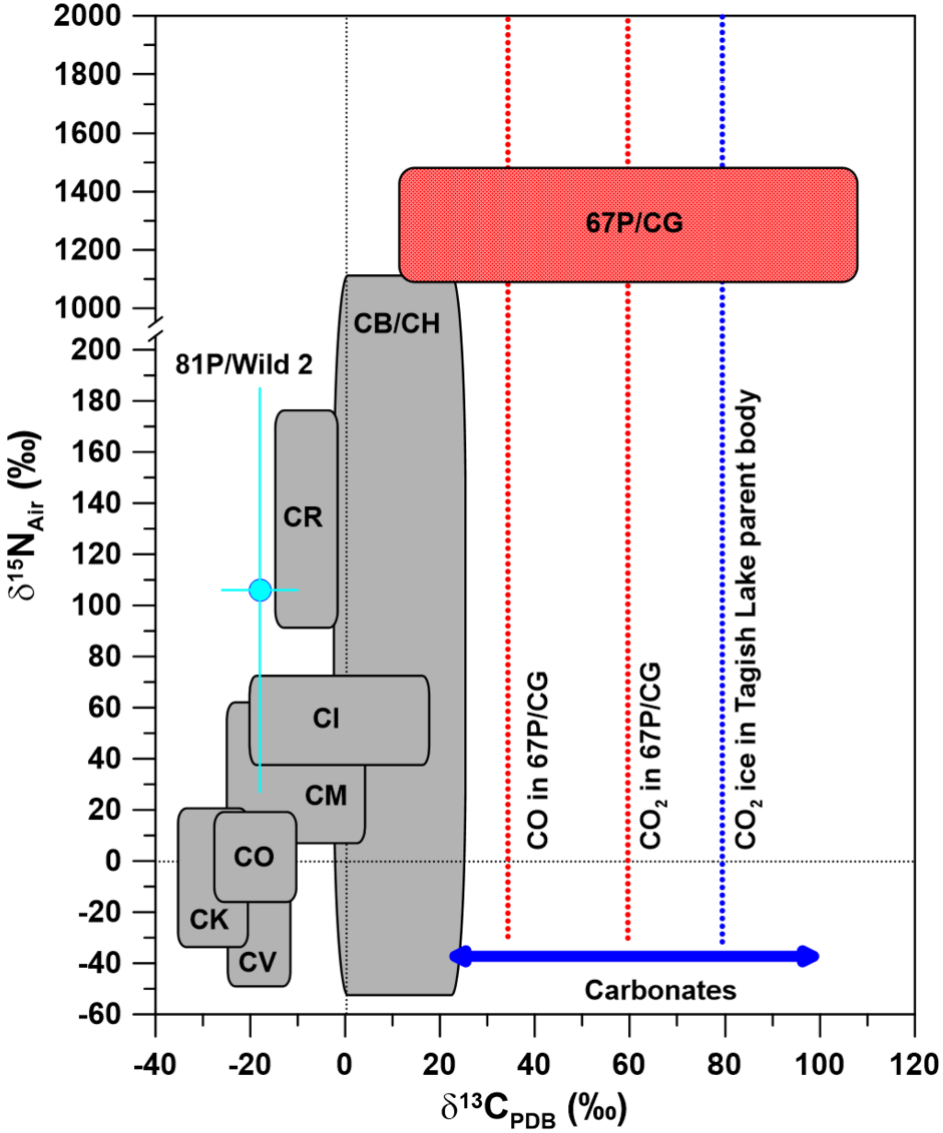
Carbon- and N-isotopic compositions, given in permil deviation from terrestrial PDB and air standards, respectively, of bulk carbonaceous chondrites, carbonates from chondrites and C-type asteroid Ryugu (C data only), comet 81P/Wild 2, and comet 67P/CG. Note the y-axis break at $\delta ^{15}\mathrm{N} \sim 200\permil $, after which the scale is different. Carbon- and N-isotopic compositions of 81P/Wild 2 represent bulk compositions inferred from residues in impact craters on Al foils from NASA’s Stardust mission. The blue arrow represents the range of $\delta ^{13}$C values of carbonates and the blue dotted line the inferred C-isotopic composition of CO_2_ ice at the location in the protoplanetary disk where the parent body of the Tagish Lake meteorite formed (Fujiya et al. 2019). The C-isotopic data for 67P/CG (red dotted lines) are for CO_2_ and CO. The width of the red box represents the $1\sigma $ uncertainty for $\delta ^{13}\mathrm{C}$ in CO_2_; the $1\sigma $ uncertainty for $\delta ^{13}\mathrm{C}$ in CO extends over the whole plot range and is not shown. The N-isotopic composition of 67P/CG is given as weighted average of $\delta ^{15}\mathrm{N}_{\mathrm{Air}}$ values of NH_3_, NO, N_2_, and HCN ($\delta ^{15}\mathrm{N}_{\mathrm{Air}}= 1290 \pm 210\permil $). Data sources: 67P/CG: Hässig et al. (2017), Altwegg et al. (2019); bulk chondrites: Alexander et al. (2012), Franchi et al. (1986), Grady and Pillinger (1990), Ivanova et al. (2008), Kerridge (1985), Pearson et al. (2006), Sugiura et al. (2000); carbonates: Fujiya et al. (2015, 2019), McCain et al. (2023); 81P/Wild 2: Stadermann et al. (2008). Errors of Wild 2 data are $1\sigma $. Figure adapted from Hoppe et al. (2018)

**Fig. 3:**
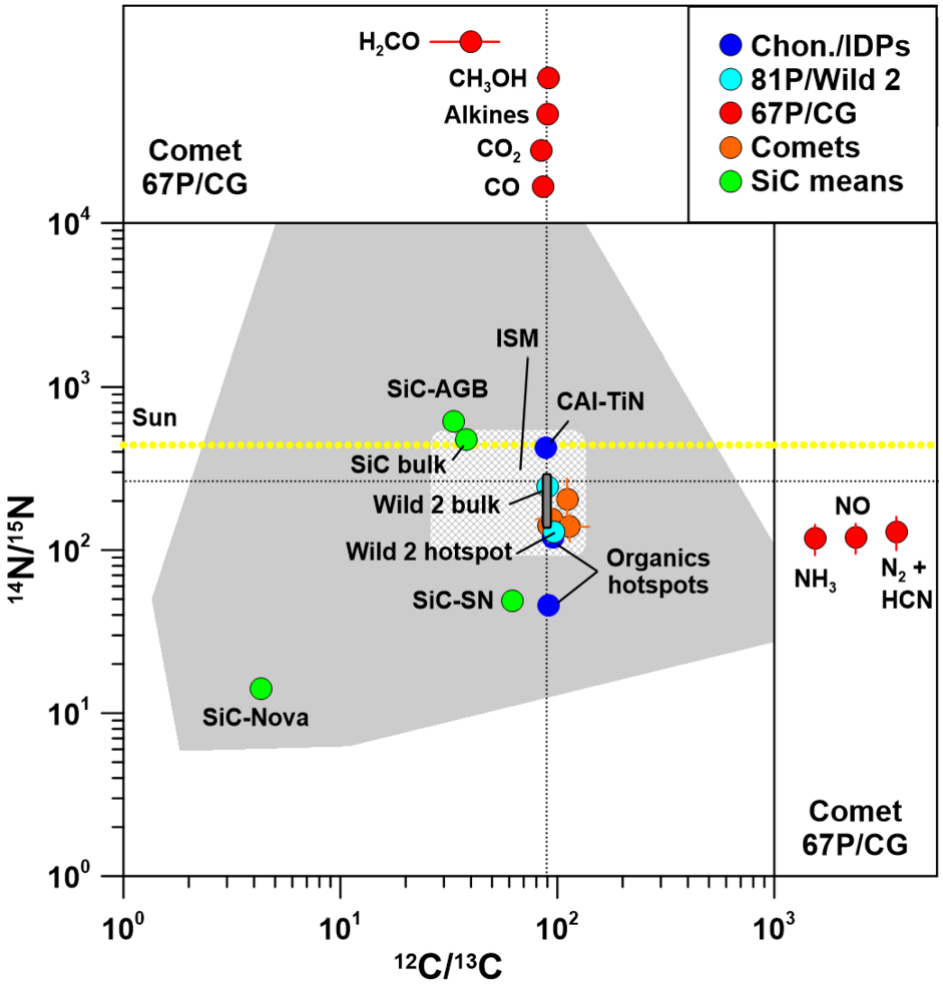
Carbon- and N-isotopic ratios of specific components in chondrites, IDPs, comets 81P/Wild 2 and 67P/CG (C or N data for different species only, upper and right panels), and other comets (spectroscopic data for CN and HCN) in comparison to presolar SiC grains (mean values for grains from AGB stars, supernovae, and novae, and for bulk SiC) and the ISM. Carbon- and N-isotopic compositions for comet 81P/Wild 2 were inferred from residues in impact craters on Al foils from NASA’s Stardust mission. Organics hotspots refer to extreme ^15^N-enrichments observed in chondrites, IDPs, asteroid Ryugu, and comet 81P/Wild 2. The medium-grey area represents the data of individual SiC grains, the light-grey hatched area those of the ISM, and the dark-grey area those for the bulk compositions of carbonaceous chondrites. The black dotted lines indicate the terrestrial PDB (C) and air (N) values. Data sources: Chondrites and IDPs: Briani et al. (2009), Floss et al. (2004), Meibom et al. (2007); 81P/Wild 2: Stadermann et al. (2008); 67P/CG: Hässig et al. (2017), Rubin et al. (2017), Altwegg et al. (2019, 2020), Müller et al. (2022); other comets: Bockelée-Morvan et al. (2015), and references therein; Sun: Marty et al. (2011); presolar SiC: Zinner (2014), and references therein; ISM: Füri and Marty (2015), Wilson (1999). Errors are $1\sigma $. Figure adapted from Hoppe et al. (2018)

Bulk carbonaceous chondrites have ^13^C/^12^C ratios that are within a few percent of the terrestrial Pee Dee Belemnite (PDB) standard (Alexander et al. 2012; Kerridge 1985; Pearson et al. 2006) (Figs. 2 and 3). Moderate enrichments in ^13^C have been observed for carbonates in carbonaceous chondrites and C-type asteroid Ryugu, with $\delta ^{13}\mathrm{C}_{\mathrm{PDB}}$ values between $+20$ and $+100\permil $ (Fig. 2; Fujiya et al. 2015, 2019; McCain et al. 2023). Carbonates in the unique Tagish Lake carbonaceous chondrite are particularly interesting because they have consistently high $\delta ^{13}\mathrm{C}_{\mathrm{PDB}}$ values of ${\sim}+70\permil $ (Fujiya et al. 2019). As inferred from reflectance spectra, the Tagish Lake meteorite is believed to be a fragment of a D-type asteroid. The Tagish Lake meteorite is very C-rich (4-5%), has a high porosity, and unusually low contents of chondrules and CAIs (Hiroi et al. 2001). Based on the specific C-isotopic ratios of carbonates, interpreted to be the signature of ^13^C-rich CO_2_ ice, and an high inferred CO_2_/H_2_O ratio as similarly observed in comets, it was argued by Fujiya et al. (2019) that the parent body of the Tagish Lake meteorite formed in the outer regions of the protoplanetary disk, beyond the orbits of Uranus and Neptune, or possibly even in the Kuiper belt. This would link the formation location of the Tagish Lake meteorite parent body to those of comets. Interestingly, measurements of the ^13^C/^12^C ratio in CO_2_ ice on Saturn’s satellite Phoebe showed strong enrichments in ^13^C, with a ^13^C/^12^C ratio of 0.053, i.e., 4.7 times the terrestrial PDB value (Clark et al. 2019). This is much higher than the ^13^C/^12^C ratio inferred for CO_2_ in the Tagish Lake meteorite and comet 67P/CG (see below). Neveu et al. (2020) interpreted the high ^13^C enrichment in CO_2_ ice from Phoebe as a result of self-shielding of CO from photodissociation in the protosolar nebula.

Carbon dioxide released from sublimated ice in 67P/CG exhibits slight enrichments in ^13^C of ∼60$\permil $ relative to PDB (Hässig et al. 2017), i.e., in Fig. 2 it plots to the right of bulk carbonaceous chondrites and comet 81P/Wild 2, but overlaps with carbonates in chondrites and C-type asteroid Ryugu (Fujiya et al. 2015, 2019; McCain et al. 2023). The C-isotopic composition of CO_2_ in 67P/CG is compatible with the $\delta ^{13}\mathrm{C}_{\mathrm{PDB}}$ value of $+80\permil $ relative to PDB inferred for CO_2_ ice in the solar nebula (Fujiya et al. 2019). The more recent C isotope measurements of carbonates in Ryugu suggest an even more ^13^C-enriched composition of CO_2_ ice with a $\delta ^{13}\mathrm{C}_{\mathrm{PDB}}$ value of up to $+100\permil $ which would still be compatible with the value measured for CO_2_ in 67P/CG when experimental uncertainties are considered. This gives further support to the idea that the outer regions of the protoplanetary disk contained ^13^C-rich CO_2_ and is in line with the conclusion of Hässig et al. (2017) who argued that the moderate ^13^C enrichment suggests formation of 67P/CG at >25 AU.

The presolar silicate abundance in the Tagish Lake meteorite is low, probably the result of aqueous alteration on the parent body (Floss and Haenecour 2016). Similar observations are made for bulk Ryugu material (Barosch et al. 2022). This contrasts with cometary CP-IDPs, matter from comet 81P/Wild 2, primitive clasts in Ryugu, and many other carbonaceous chondrites which have much higher presolar silicate abundances (Floss and Haenecour 2016; Nguyen et al. 2022b). This illustrates the large diversity of small planetary bodies from the outer parts of the protoplanetary disk and the different degrees of alteration they experienced, and possibly also heterogeneities among the starting materials. On the other hand, Tagish Lake has an abundance of carbonaceous presolar grains (Riebe et al. 2018) comparable to the tens of ppm found in other carbonaceous chondrites (Davidson et al. 2014) and bulk Ryugu (Barosch et al. 2022). It would be interesting to see how much carbonaceous presolar grains would be present in 67P/CG and whether the tens of ppm seen in primitive meteorites, including those from outer regions in the protoplanetary disk, are representative for the solar nebula. Unfortunately, there are no reliable estimates on the abundances of carbonaceous presolar grains in cometary IDPs and comet 81P/Wild 2, although presolar SiC abundances appear to be clearly lower than those of presolar silicates. As discussed by Hoppe et al. (2018), C-isotopic signatures of carbonaceous presolar grains are not expected to be seen in the C-isotopic compositions of CO and CO_2_ from comet 67P/CG but may be recognizable in the refractory component of 67P/CG if concentrations of carbonaceous presolar grains, which exhibit large C-isotopic anomalies (Fig. 3), would be close to 1%, i.e., much higher than in chondrites.

The C-isotopic compositions of C_2_, CN, and HCN in the comae of several comets determined by spectroscopic measurements (Fig. 3; Bockelée-Morvan et al. 2015, and references therein) are largely compatible (within a few percent) with those of CO_2_ in 67P/CG (Hässig et al. 2017) and carbonates in carbonaceous chondrites and Ryugu (Fujiya et al. 2015, 2019; McCain et al. 2023) when uncertainties are considered. The same is true for the C-isotopic composition of CO, alkanes, and methanol from 67P/CG (Fig. 3; Rubin et al. 2017; Altwegg et al. 2020). Formaldehyde (H_2_CO) from 67P/CG, on the other hand, exhibits a strong enrichment in ^13^C of about a factor of 2 relative to solar (Fig. 3; Altwegg et al. 2020). As discussed in Altwegg et al. (2020), these ^13^C enrichments are qualitatively in line with observations of formaldehyde in the ISM and in massive young stellar objects. For meteorites similarly high ^13^C enrichments are observed only in presolar grains (see Hoppe et al. 2018). The different C-isotopic ratios of methanol and formaldehyde in 67P/CG suggest different formation pathways for these two molecules (see also section on oxygen below).

### Nitrogen

On a bulk scale, N-isotopic variations of primitive Solar System materials available for laboratory studies are generally much larger than the few percent observed for carbon. As outlined in the review by Hoppe et al. (2018), bulk N-isotopic compositions of chondrites are typically within a few percent of terrestrial air, but certain groups of chondrites (CR, CB/CH) and CP-IDPs of presumably cometary origin also exhibit strong ^15^N enrichments of up to a factor of >2 (Alexander et al. 2012; Franchi et al. 1986; Grady and Pillinger 1990; Ivanova et al. 2008; Kerridge 1985; Pearson et al. 2006; Sugiura et al. 2000; Floss et al. 2006) (Figs. 2 and 3). On a micrometer scale, ^15^N can be strongly enriched relative to the surrounding material by up to a factor of 6 in organics of chondrites, IDPs, and C-type asteroid Ryugu (Alexander et al. 2007; Briani et al. 2009; Floss et al. 2004; Remusat et al. 2022) (Fig. 3). The Sun has a ^14^N/^15^N ratio about 40% higher than that of terrestrial air, as inferred from measurements of the solar wind (Fig. 3; Marty et al. 2011), i.e., relative to this reference observed ^15^N enrichments of planetary materials are even more extreme.

Nitrogen-isotopic compositions of NH_3_, NO, and N_2_ + HCN have been derived for 67P/CG (Altwegg et al. 2019). NH_3_ and NO have ^14^N/^15^N ratios of $118 \pm 25$ ($\delta ^{15}\mathrm{N}_{\mathrm{Air}} = 1310 \pm 490\permil $) and $120 \pm 25$ ($\delta ^{15}\mathrm{N}_{\mathrm{Air}} = 1170 \pm 470\permil $), respectively; because of isobaric interferences with major volatile species in ROSINA mass spectra, the N-isotopic compositions of N_2_ and HCN could be determined only together when investigating their fragment ions ${}^{14}\mathrm{N}^{+}$ and ${}^{15}\mathrm{N}^{+}$ (with contributions of 68% N_2_ and 32% HCN after subtraction of the corresponding contribution of NH_3_ and NO), which yielded ${}^{14}\mathrm{N}/{}^{15}\mathrm{N} = 130 \pm 30$ ($\delta ^{15}\mathrm{N}_{\mathrm{Air}} = 1090 \pm 480\permil $) (Altwegg et al. 2019). This indicates that the comet doesn’t contain two separate reservoirs of nitrogen based on different isotope ratios in both atomic and molecular nitrogen discussed earlier (Hily-Blant et al. 2017). These ^14^N/^15^N ratios fall at the ^15^N-rich end of values inferred spectroscopically for HCN, CN, and NH_2_ from other comets (Bockelée-Morvan et al. 2015) and the most extreme bulk compositions of CB/CH chondrites (Figs. 2 and 3), and plot between those of bulk compositions of meteorites from the major carbonaceous chondrite groups on one side (lower $\delta ^{15}\mathrm{N}$ or higher ^14^N/^15^N, respectively), and organic hotspots from chondrites, IDPs, asteroid Ryugu, and comet 81P/Wild 2 as well as presolar SiC grains from supernovae and novae on the other side (lower ^14^N/^15^N; Figs. 2 and 3). The N- (and C-) isotopic compositions of the various molecular species from 67P/CG lie also well within the ranges determined for molecules in the ISM (Fig. 3; Wilson 1999; Milam et al. 2005; Füri and Marty 2015).

### Oxygen

**Fig. 4:**
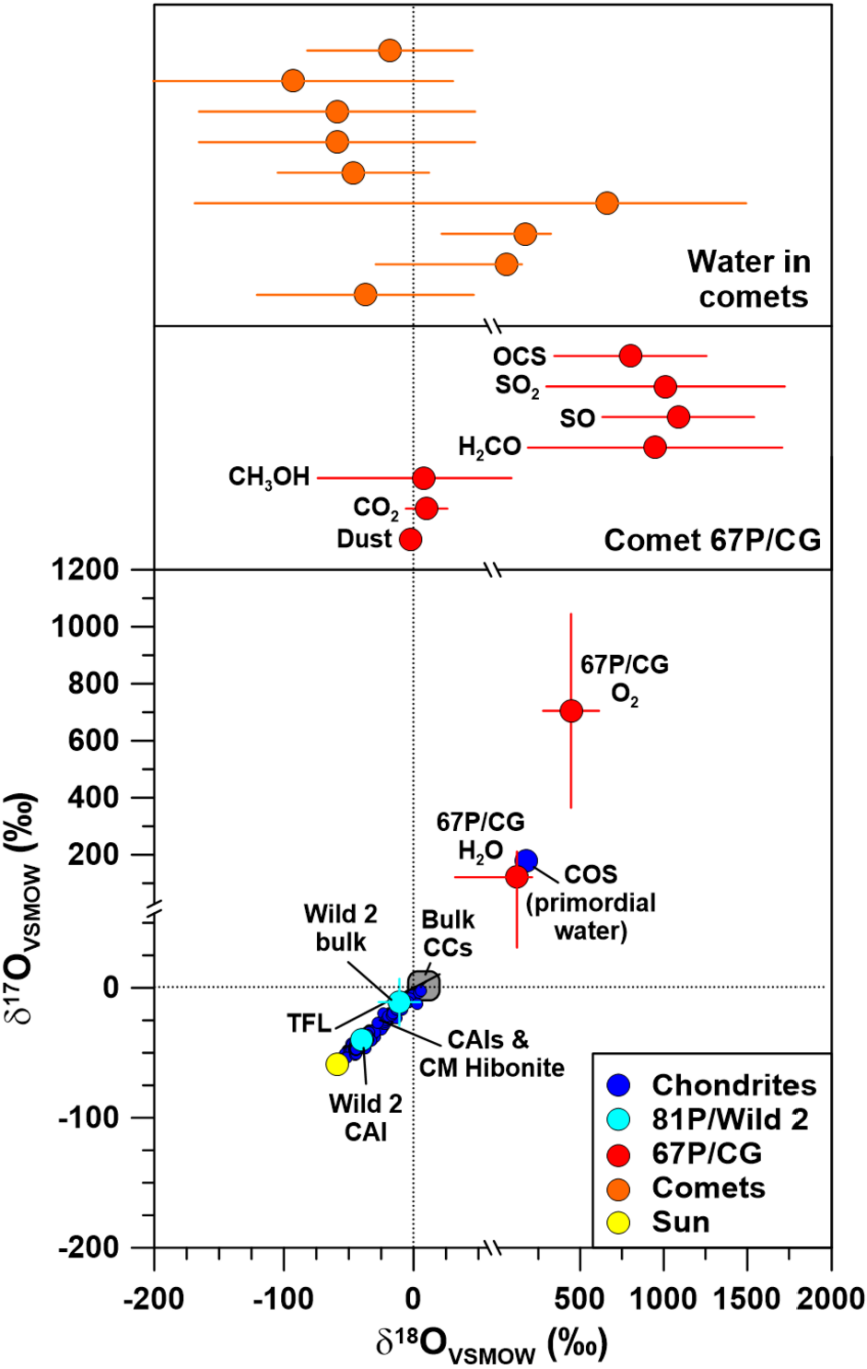
Oxygen-isotopic compositions, given as permil deviation from terrestrial VSMOW, of various components of carbonaceous chondrites, comets 81P/Wild 2 and 67P/CG (for dust, CO_2_, CH_3_OH, H_2_CO, SO, SO_2_, and OCS $\delta ^{18}\mathrm{O}$ only, middle panel), other comets (ground-based spectroscopic observations of water, $\delta ^{18}\mathrm{O}$ only, upper panel), and the Sun. Note the x- and y-axis breaks at $\delta ^{17,18}\mathrm{O} = 50\permil $, after which the scales are different. Data for Wild 2 bulk composition represent residues in impact craters on Al foils from NASA’s Stardust mission. The bulk O-isotopic composition of C-type asteroid Ryugu (not shown) is compatible with CI chondrites (Yokoyama et al. 2023). COS: cosmic symplectite, assumed to represent primordial water in the solar nebula. TFL: Terrestrial fractionation line. Data sources: Chondrites: Aléon et al. (2002), Clayton (2004), Fagan et al. (2002), Krot et al. (2002), Liu et al. (2009), Kööp et al. (2016a,b), Lodders and Fegley (1998), Sakamoto et al. (2007); Sun: McKeegan et al. (2011); 81P/Wild 2: McKeegan et al. (2006), Stadermann et al. (2008); 67P/CG: Hässig et al. (2017), Paquette et al. (2018), Schroeder et al. (2019), Altwegg et al. (2020), Müller et al. (2022); water in comets: Biver et al. (2007), Bockelée-Morvan et al. (2015), and references therein. Errors are $1\sigma $. Figure adapted from Hoppe et al. (2018)

Oxygen-isotopic compositions of bulk chondrites and matter from comet 81P/Wild 2 are within a few permil of the terrestrial VSMOW standard (Fig. 4). This also holds for C-type asteroid Ryugu which has a bulk O-isotopic composition compatible with that of CI chondrites (Yokoyama et al. 2023). Much larger O isotope anomalies are evident for certain components in chondrites, e.g., calcium-aluminum-rich inclusions (CAIs), hibonite grains, cosmic symplectite in the Acfer 094 meteorite (COS; Sakamoto et al. 2007), silica-rich grains embedded in organic matter from the Murchison meteorite (Aléon et al. 2005), and presolar grains (Zinner 2014). CAIs and hibonites exhibit enrichments in ^16^O of up to 60$\permil $, the value inferred for O in the Sun (McKeegan et al. 2011), along a line with slope ∼1 in an O three-isotope-representation (Fig. 4; Aléon et al. 2002; Fagan et al. 2002; Krot et al. 2002; Liu et al. 2009; Kööp et al. 2016a,b). The COS phase shows enrichments in ^17^O and ^18^O of about 180$\permil $ (Sakamoto et al. 2007), i.e., it falls on the extension of the slope 1 line defined by CAIs and hibonites to the ^16^O-poor side in the O three-isotope representation (Fig. 4). COS consists of aggregates of nanocrystalline iron sulfide and magnetite. Its ^17^O and ^18^O enrichments are interpreted to be the result of oxidation of Fe,Ni metal and sulfides by primordial ^16^O-poor water in the Solar System (Sakamoto et al. 2007). A comparison with cometary water is thus of particular importance.

Ground-based spectroscopic observations of several comets have provided ^16^O/^18^O, but not ^16^O/^17^O ratios of H_2_O (Bockelée-Morvan et al. 2015, and references therein). ^16^O/^18^O ratios with errors of ∼15% or less are between $425 \pm 55$ and $530 \pm 60$; the weighted average of ^16^O/^18^O of H_2_O of the 6 measurements listed in Bockelée-Morvan et al. (2015) is $495 \pm 19$, i.e., $\delta ^{18}\mathrm{O}_{\mathrm{VSMOW}} = 7 \pm 19\permil $. The average $\delta ^{18}\mathrm{O}_{\mathrm{VSMOW}}$ value is fully compatible with VSMOW but clearly lower than the value for COS. However, we note that 3 of the 9 cometary measurements gave $\delta ^{18}\mathrm{O}$ values which are compatible with that of COS if $2\sigma $ errors are considered (Fig. 4).

Oxygen-isotopic ratios of H_2_O in comet 67P/CG were measured by ROSINA (Altwegg et al. 2015; Schroeder et al. 2019; Müller et al. 2022). The preliminary data for H_2_O of Altwegg et al. (2015) turned out to suffer from an insufficient consideration of detector aging; however, the recent development of a more sophisticated detector aging model takes care of this and provides reliable O isotope data for H_2_O, with ${}^{16}\mathrm{O}/{}^{17}\mathrm{O}= 2347 \pm 191$ (Müller et al. 2022), or $\delta ^{17}\mathrm{O}_{\mathrm{VSMOW}} = 121 \pm 91\permil $, and ${}^{16}\mathrm{O}/{}^{18}\mathrm{O} = 445 \pm 35$ (Schroeder et al. 2019), or $\delta ^{18}\mathrm{O}_{\mathrm{VSMOW}} = 122 \pm 90\permil $ (Fig. 4). The significance of these anomalies is $1.3\sigma $ (^17^O) and $1.4\sigma $ (^18^O), respectively. Oxygen isotope data of H_2_O were also obtained with the MIRO (Microwave Instrument for Rosetta Orbiter) instrument (Biver et al. 2019). The ^18^O/^17^O ratio of $5.6 \pm 0.8$ determined by MIRO for H_2_O is compatible with the ROSINA result. Interestingly, the ^17^O- and ^18^O-enriched composition of cometary water is compatible with that of meteoritic COS, which is interpreted to represent primordial water in the solar nebula (Sakamoto et al. 2007; see above). A popular model to account for enrichments of the heavy oxygen isotopes in primordial water is isotope-selective self-shielding by UV photodissociation of CO in the solar nebula (Clayton 2002; Yurimoto and Kuramoto 2004); predicted enrichments of ^17^O and ^18^O in atomic oxygen and water relative to terrestrial water are in a range of 5-20% (Yurimoto and Kuramoto 2004), in good agreement with the data for water in 67P/CG and meteoritic COS. However, other molecules from 67P/CG exhibit much larger O isotope fractionations and may therefore need another explanation, which then may also be applicable to water (Altwegg et al. 2020).

Besides water, ^16^O/^18^O has also been measured in CO_2_ (Hässig et al. 2017), O_2_ (together with ^16^O/^17^O), methanol, formaldehyde, SO, SO_2_, and OCS in the coma of 67P/CG (Altwegg et al. 2020). The combined results reveal strong variations in the oxygen isotopes among the different O-bearing species of the same comet (Fig. 4). While CO_2_ and methanol have ^16^O/^18^O ratios compatible with the terrestrial VSMOW standard and meteoritic bulk compositions, O_2_ shows an enrichment in ^18^O (and ^17^O) of about a factor 1.5. It is interesting to note that in an oxygen three-isotope representation O_2_ plots roughly on the extension of the slope 1 array defined by the Sun, refractory components from meteorites, COS, and water in 67P/CG (Fig. 4). Even larger ^18^O enrichments of a factor of about 2 are observed for formaldehyde, SO, SO_2_, and OCS from 67P/CG. For meteorites, similarly high ^18^O enrichments are only evident for presolar grains and silica-rich grains embedded in organics (see Hoppe et al. 2018).

It was argued that the stronger enrichments of the heavy O isotopes in O_2_ compared to water rules out production of molecular oxygen in 67P/CG by radiolysis from water, by dismutation of H_2_O_2_, or by collisions of energetic water ions with the cometary surface (Altwegg et al. 2020). As discussed in Altwegg et al. (2020), the similar O-isotopic compositions of CO_2_ and methanol could be explained if methanol is a product of CO from successive hydrogenation on cold grains, and CO_2_ the product of oxygenation of CO. The different O-isotopic signatures of methanol and formaldehyde suggest that these two species do not share the same formation path, a conclusion that is also supported by the C-isotopic ratios (see above). The ^18^O-enriched composition of S-bearing species in 67P/CG is in line with measurements of OCS in the ISM, but that of formaldehyde disagrees with the higher-than-solar ^16^O/^18^O ratio inferred for formaldehyde in the ISM (Altwegg et al. 2020). Numerical models of oxygen fractionation in cold and dense molecular clouds predict quite similar variations for ^16^O/^18^O ratios as observed in 67P/CG, except for formaldehyde (Loison et al. 2019), and provide a clear link to a molecular cloud origin of the ices in 67P/CG with very limited O isotope exchange.

### Chlorine and Bromine

As reported by Dhooghe et al. (2017), the main halogen-bearing species in the coma of comet 67P/CG are HF, HCl, and HBr. Relative to oxygen, bulk abundances are $\text{F/O} \sim 8.9 \times 10^{-5}$, $\text{Cl/O} \sim 1.2 \times 10^{-4}$, and $\text{Br/O} \sim 2.5 \times 10^{-6}$. These values are relatively similar to the values of $\text{F/O} \sim 1.0 \times 10^{-4}$, $\text{Cl/O} \sim 6.8 \times 10^{-4}$, and $\text{Br/O} \sim 1.4 \times 10^{-6}$ determined for CI chondrites, and $\text{F/O} \sim 6.8 \times 10^{-5}$ and $\text{Cl/O} \sim 5.9 \times 10^{-4}$ measured in the solar photosphere (no Br data) (Lodders et al. 2009). Recently, other Cl-bearing species besides HCl were identified in the coma of 67P/CG, namely, NH_4_Cl (Altwegg et al. 2020) and CH_3_Cl (Fayolle et al. 2017). The isotopic ratios determined by Dhooghe et al. (2017) for ^37^Cl/^35^Cl of $0.29 \pm 0.02$ and ^81^Br/^79^Br of $0.95 \pm 0.07$ are compatible with the ratios on Earth and in chondrites within the experimental $1.5 \sigma $ uncertainties (Table 1). Dhooghe et al. (2017) argued that these observations point to an origin of hydrogen halides by molecular cloud chemistry and incorporation of frozen hydrogen halides on dust grains into comets. In a follow-up work Dhooghe et al. (2021) extended the analysis of 67P/CG data from Dhooghe et al. (2017), which was based on data from one month only, to the entire mission. Detailed data analysis of Dhooghe et al. (2021) suggests the presence of an yet unknown Cl-bearing species, besides HCl, CH_3_Cl, and NH_4_Cl. The ^37^Cl/^35^Cl ratio determined by Dhooghe et al. (2021) is 0.336 ± 0.017 which is slightly higher ($1.8\sigma $) than the value of Dhooghe et al. (2017) and agrees with the terrestrial and chondritic value within $1\sigma $.

**Fig. 5:**
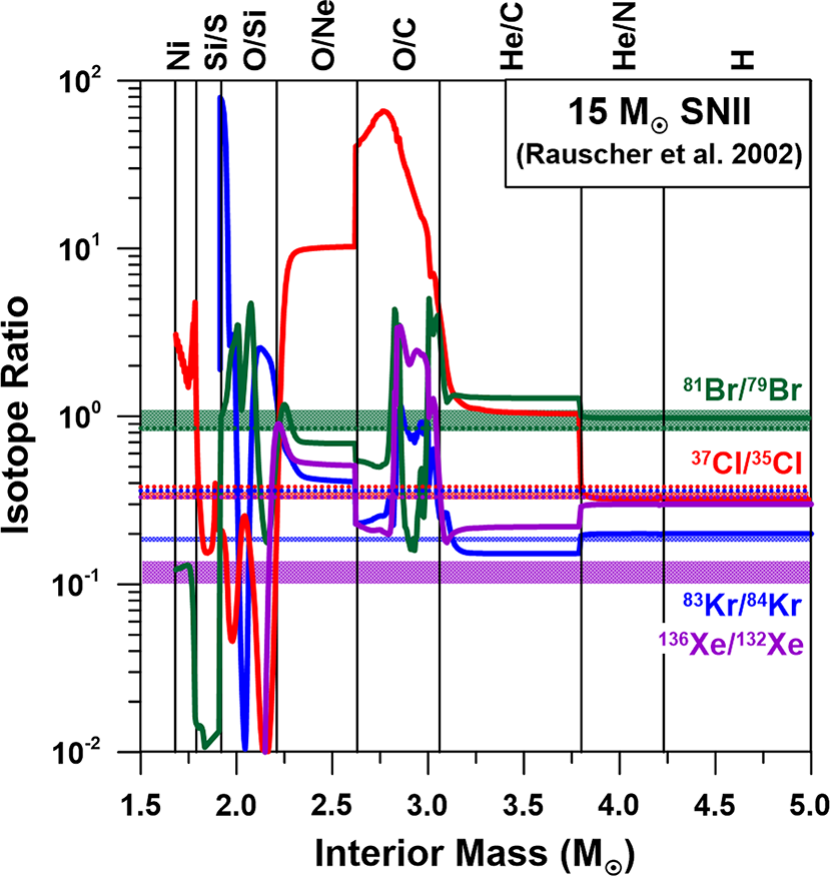
Profiles of selected isotopic ratios of volatile elements Cl, Br, Kr, and Xe in the interior of a $15~\text{M}_{\odot}$ Type II SN according to Rauscher et al. (2002). This model provided good fits for the isotopic compositions of many elements in presolar grains from SNe (e.g., Hoppe et al. 2010) and for the S-isotopic compositions of volatile species from 67P/CG (Hoppe et al. 2018). The names of SN zones are indicated at the top of the figure and follow the nomenclature of Meyer et al. (1995). Isotopic compositions of the total ejecta are indicated by horizontal dotted lines. The horizontal colored bands represent the isotopic ratios measured for comet 67P/CG. While the ^37^Cl/^35^Cl and ^81^Br/^79^Br ratios of 67P/CG are roughly compatible with those predicted for the total SN ejecta, ^83^Kr/^84^Kr and ^136^Xe/^132^Xe of 67P/CG are significantly lower

Following the discussion in Hoppe et al. (2018) on a potential late SN contribution to the formation location of 67P/CG, as suggested by the Si- and S-isotopic data of 67P/CG, we can compare ^37^Cl/^35^Cl and ^81^Br/^79^Br ratios in 67P/CG with those predicted for the total ejecta in $15~\text{M}_{\odot}$ and $25~\text{M}_{\odot}$ Type II SNe (Rauscher et al. 2002). We find satisfactory agreements within experimental uncertainties for ^37^Cl/^35^Cl in the 15 and $25~\text{M}_{\odot}$ and for ^81^Br/^79^Br in the $15~\text{M}_{\odot}$ SN models (Fig. 5). However, the fact that Cl- and Br-isotopic ratios are about solar, makes them not very diagnostic with respect to the scenario of a late SN contribution to the formation site of 67P/CG.

### Krypton

**Fig. 6:**
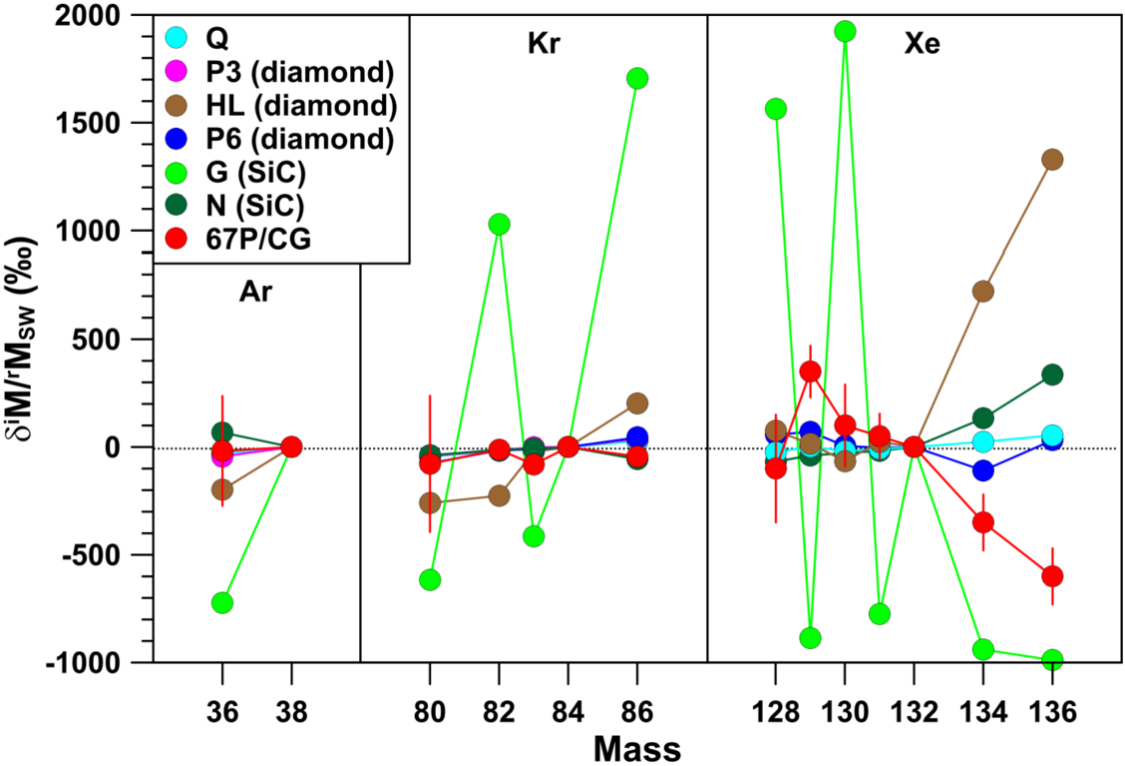
Argon-, Kr-, and Xe-isotopic compositions, given as permil deviation from solar wind Ar, Kr, and Xe, respectively, of different components in meteorites and of Ar, Kr, and Xe released from sublimation of ice in 67P/CG. $\delta ^{\mathrm{i}}\mathrm{M}/{}^{\mathrm{r}}\mathrm{M}= [({}^{\mathrm{i}}\mathrm{M}/{}^{\mathrm{r}}\mathrm{M})/({}^{\mathrm{i}} \mathrm{M}/{}^{\mathrm{r}}\mathrm{M}) _{\mathrm{SW}}- 1] \times 1000$; $\mathrm{M} = \text{Ar, Kr, Xe}$; $\mathrm{i} = \mbox{mass number on $x$-axis}$; $\mathrm{r} = 38$ (Ar), 84 (Kr), 132 (Xe). Components P3, HL, and P6 are contained in presolar diamonds, G and N in presolar SiC. Values for ^80^Kr/^84^Kr and ^86^Kr/^84^Kr of the G component represent the means of the lowest and highest ratios inferred from presolar SiC. Data sources: Balsiger et al. (2015), Marty et al. (2017), Rubin et al. (2018), Ott (2014). Errors are shown for 67P/CG only and are $1\sigma $

Meteorites contain trapped noble gases (implanted solar wind and the planetary components Q, P3, HL, P6, G, N) and those produced in situ by spallation or radioactive decay. Except Q, all planetary noble gas components are considered presolar. For a detailed review see Ott (2014). In Hoppe et al. (2018) we compared the isotope data of Ar and Xe in meteorites with those for 67P/CG. It was found that the ^36^Ar/^38^Ar ratio of 67P/CG (Balsiger et al. 2015) is compatible with that of the Earth, solar wind, and all trapped planetary components, except the G component (Fig. 6). The most pronounced signature of Xe are large depletions in the heavy isotopes ^134^Xe and ^136^Xe relative to ^132^Xe (Marty et al. 2017) (Fig. 6). The Xe-isotopic pattern of 67P/CG does not agree with any of the patterns of the trapped planetary noble gas components but can be matched relatively well by a mixture of s-process Xe and two r-process endmember compositions identified by Gilmour and Turner (2007). In the following we will expand the comparison to the Kr-isotopic composition which, as for Ar and Xe, was determined by ROSINA (Rubin et al. 2018).

Like Ar and Xe, Kr was released by sublimation of ice from 67P/CG. It was shown that the abundances of the noble gases correlate rather well with the abundance of N_2_ (Rubin et al. 2018). By combining noble gas/N_2_ ratios with the N_2_/H_2_O ratio measured close to perihelion, bulk concentrations of the noble gases were calculated after correction for the different outgassing velocities of the different species. The Kr/H_2_O ratio was determined to $(4.9 \pm 2.2) \times 10^{-7}$, compared to $(5.8 \pm 2.2) \times 10^{-6}$ and $(2.4 \pm 1.1) \times 10^{-7}$ for the Ar/H_2_O and Xe/H_2_O ratios, respectively (Rubin et al. 2018).

The overall isotope pattern of Kr in 67P/CG is qualitatively similar to those of the solar wind and the trapped planetary components Q, P3, P6, and N, but distinct from those of the HL and G components (Fig. 6). Within $2\sigma $, ^80^Kr/^84^Kr, ^82^Kr/^84^Kr, and ^86^Kr/^84^Kr ratios agree with those of the solar wind, whereas the ^83^Kr/^84^Kr ratio is slightly, but with ${>}2\sigma $ significance, lower than the ratio in the solar wind, with $\delta ^{83}\mathrm{Kr}_{\mathrm{SW}} = -81 \pm 34\permil $ (Rubin et al. 2018) (Fig. 6). It was pointed out by Rubin et al. (2018) that the Kr-isotopic pattern of 67P/CG can be reasonably well fitted by adding 5% G-Kr to N-Kr and by considering the low ^86^Kr/^84^Kr ratio of the weak s-process (see Gilmour 2010) for G-Kr. Note that the $\delta ^{86}$Kr_SW_ value of the G component shown in Fig. 6 is not representative of the weak s-process but represents the mean of the lowest and highest ratio of the G component inferred from presolar SiC (Ott 2014). Clearly, the Kr isotope pattern of 67P/CG adds to the complex picture emerging from noble gases in 67P/CG, which demonstrates that the processes that led to Solar System formation are far from being fully understood.

Like for the volatiles CO_2_, S, Ar, Xe, Cl, and Br in comet 67P/CG discussed in Hoppe et al. (2018) and above, we can compare the Kr-isotopic composition predicted for the total ejecta of a single SN with that of 67P/CG. If we consider the $15~\text{M}_{\odot}$ and $25~\text{M}_{\odot}$ Type II SN models of Rauscher et al. (2002), predicted Kr-isotopic patterns differ significantly from what is observed in 67P/CG; the SN ejecta have ^84^Kr-normalized ratios that are higher by factors of 1.2 to 3.7 (Fig. 5). As similarly concluded for CO_2_, Xe (Fig. 5), Cl, and Br, the Kr-isotopic data of 67P/CG don’t provide further support for the hypothetical late injection of matter from a nearby SN to the formation site of 67P/CG.

## Summary and Conclusions

ESA’s Rosetta mission to comet 67P/CG has provided a wealth of isotope data that allow to get new insights into the origin of the Solar System. As of March 2023, isotope data are available for H, C, N, O, Si, S, Cl, Ar, Br, Kr, and Xe in 67P/CG. Here, we have reviewed new isotope data of H, C, N, O, Cl, Br, and Kr and discussed them in the context of respective data obtained for meteorites and IDPs, and samples from C-type asteroid Ryugu which shows a close relationship to CI chondrites. This complements the review paper of Hoppe et al. (2018). The most important findings and conclusions from the new isotope data can be summarized as follows:

1. The D/H ratio of methanol in 67P/CG is more than an order of magnitude higher than those of H_2_O and H_2_S which are enriched in D by factors of about 3-4 relative to terrestrial water; it is also higher than D/H in any primitive planetary materials available for studies in terrestrial laboratories but compatible with D/H ratios of methanol observed in cold prestellar cores and low-mass protostars. Refractory organics, alkanes, and NH_3_ in 67P/CG are 2-5× more D-rich than water in 67P/CG and overlap with D/H ratios of meteoritic organics.
2. Carbonates in the Tagish Lake meteorite, CM2 chondrites, and C-type asteroid Ryugu show ^13^C enrichments of up to ∼10% relative to the terrestrial PDB standard which is compatible with what was inferred for CO_2_ in 67P/CG. Based on the ^13^C enrichments of carbonates and a high CO_2_/H_2_O ratio as similarly observed in comets, it was argued that the parent body of the Tagish Lake meteorite, presumably a D-type asteroid, formed in the outer reaches of the protoplanetary disk, i.e., in the formation region of comets. This provides evidence for a large diversity or continuum of small planetary bodies from the outer parts of the protoplanetary disk.
3. While CO, CO_2_, alkines, and methanol in 67P/CG have C-isotopic compositions largely compatible with solar C, formaldehyde exhibits a strong enrichment in ^13^C of about a factor of 2. This ^13^C enrichment is in line with measurements for the ISM; for meteorites, IDPs, and C-type asteroid Ryugu, on the other hand, similarly high ^13^C enrichments are observed only in presolar grains.
4. Relative to terrestrial air, NH_3_, NO, and N_2_ + HCN in 67P/CG exhibit ^15^N enrichments by more than a factor of 2. No N-isotopic heterogeneities are observed, pointing to a single N reservoir for these molecular species, contrary to previous suggestions. The average $\delta ^{15}\mathrm{N}_{\mathrm{Air}}$ value of ${\sim }1300\permil $ (^14^N/^15^N ∼ 120) is at the upper end of bulk compositions of carbonaceous chondrites and of what was observed spectroscopically for HCN, CN, and NH_2_ from other comets. Meteorites, IDPs, and Ryugu contain submicrometer- and micrometer-sized inclusions (organics, presolar grains) that show ^15^N-enrichments that can be even higher than those of the volatile component of 67P/CG.
5. The Rosetta mission provided for the first time abundance data for all three O isotopes in cometary water. The abundances of ^17^O and ^18^O in H_2_O of 67P/CG are enhanced by ∼12%, compared to bulk chondrites (with $1.3 \sigma $ and $1.4 \sigma $ significance, respectively). These enrichments in ^17^O and ^18^O are compatible with those of meteoritic COS, interpreted to represent primordial water in the solar nebula.
6. Oxygen isotope abundances have been shown to differ among various O-bearing species including H_2_O, CO_2_, O_2_, methanol, formaldehyde, SO, SO_2_, and OCS in 67P/CG, with ^18^O enrichments up to a factor of 2 in formaldehyde and all S-bearing species. For meteorites, IDPs, and Ryugu, similarly high ^18^O enrichments are only evident for presolar grains and silica-rich grains embedded in organics. Numerical models of oxygen fractionation in cold and dense molecular clouds predict quite similar variations as observed in 67P/CG and provide a clear link to a molecular cloud origin of the ices in 67P/CG with very limited O isotope exchange.
7. Identified halogen-bearing species in 67P/CG are HF, HCl, CH_3_Cl, NH_4_Cl, and HBr. Chlorine- and Br-isotopic ratios are fully compatible with those on Earth and in chondrites.
8. The Kr-isotopic pattern of 67P/CG is similar to that of the solar wind and trapped planetary components Q, P3, P6, and N found in meteorites. The ^83^Kr/^84^Kr ratio, however, differs from solar wind Kr by more than $2\sigma $. The Kr isotopic pattern is best explained by a mixture of the planetary components G and N (found in presolar SiC). This adds to the complexity emerging from the isotopic patterns of the other noble gases in 67P/CG.
9. No supporting evidence was found for the scenario of a contribution of a nearby SN to the formation site of 67P/CG, as suggested by the Si and S isotope data, from the isotopic compositions of volatiles Cl, Br, and Kr in 67P/CG.

Most of the currently available isotope data for comet 67P/CG are for the volatile component measured by ROSINA. Isotope data for the refractory component would be important to complement our current knowledge of isotopic compositions of 67P/CG and their context to meteorites. To date this is limited to H-, O-, and S-isotopic data for a few dust grains (Paquette et al. 2017, 2018, 2021), as well as Si isotope data obtained from sputtered Si neutrals, produced by bombardment of the cometary surface by solar wind protons (Rubin et al. 2017; Wurz et al. 2015). Because comprehensive C- and O-isotopic data for a large number of carbonaceous and O-rich dust grains are not available, it is currently not possible to estimate the fraction of presolar (stardust) grains in the refractory component of 67P/CG.
